# Antibody Profiling of Pan-Cancer Viral Proteome Reveals Biomarkers for Nasopharyngeal Carcinoma Diagnosis and Prognosis

**DOI:** 10.1016/j.mcpro.2024.100729

**Published:** 2024-02-01

**Authors:** Te Liang, Hao Chen, Lei Liu, Yongqiang Zheng, Zhaoen Ma, Ling Min, Jiahui Zhang, Lianfu Wu, Jie Ma, Zexian Liu, Qingfeng Zhang, Kai Luo, Di Hu, Tianxing Ji, Xiaobo Yu

**Affiliations:** 1 Beijing Key Laboratory for Forest Pest Control, Beijing Forestry University, Beijing, China; 2 State Key Laboratory of Oncology in South China, Collaborative Innovation Center for Cancer Medicine, Guangdong Key Laboratory of Nasopharyngeal Carcinoma Diagnosis and Therapy, Sun Yat-Sen University Cancer Center, Guangzhou, China; 3 State Key Laboratory of Proteomics, Beijing Proteome Research Center, National Center for Protein Sciences-Beijing (PHOENIX Center), Beijing Institute of Lifeomics, Beijing, China; 4 Otolaryngological department, The Second Affiliated Hospital of Guangzhou Medical University, Guangzhou, China; 5 Department of Laboratory Medicine, Affiliated Cancer Hospital & Institute of Guangzhou Medical University, Guangzhou, China; 6 ProteomicsEra Medical Co., Ltd., Beijing, China; 7 Clinical Laboratory Medicine Department, The Second Affiliated Hospital of Guangzhou Medical University, Guangzhou, China

**Keywords:** protein microarray, nasopharyngeal carcinoma, biomarkers, antiviral antibodies, early detection

## Abstract

Diagnosing, predicting disease outcome, and identifying effective treatment targets for virus-related cancers are lacking. Protein biomarkers have the potential to bridge the gap between prevention and treatment for these types of cancers. While it has been shown that certain antibodies against EBV proteins could be used to detect nasopharyngeal carcinoma (NPC), antibodies targeting are solely a tiny part of the about 80 proteins expressed by the EBV genome. Furthermore, it remains unclear what role other viruses play in NPC since many diseases are the result of multiple viral infections. For the first time, this study measured both IgA and IgG antibody responses against 646 viral proteins from 23 viruses in patients with NPC and control subjects using nucleic acid programmable protein arrays. Candidate seromarkers were then validated by ELISA using 1665 serum samples from three clinical cohorts. We demonstrated that the levels of five candidate seromarkers (EBV-BLLF3-IgA, EBV-BLRF2-IgA, EBV-BLRF2-IgG, EBV-BDLF1-IgA, EBV-BDLF1-IgG) in NPC patients were significantly elevated than controls. Additional examination revealed that NPC could be successfully diagnosed by combining the clinical biomarker EBNA1-IgA with the five anti-EBV antibodies. The sensitivity of the six-antibody signature at 95% specificity to diagnose NPC was comparable to the current clinically-approved biomarker combination, VCA-IgA, and EBNA1-IgA. However, the recombinant antigens of the five antibodies are easier to produce and standardize compared to the native viral VCA proteins. This suggests the potential replacement of the traditional VCA-IgA assay with the 5-antibodies combination to screen and diagnose NPC. Additionally, we investigated the prognostic significance of these seromarkers titers in NPC. We showed that NPC patients with elevated BLLF3-IgA and BDLF1-IgA titers in their serum exhibited significantly poorer disease-free survival, suggesting the potential of these two seromarkers as prognostic indicators of NPC. These findings will help develop serological tests to detect and treat NPC in the future.

## Highlights

• Determined the serum antibody profiles of NPC patients using viral proteome arrays.
• Examined the association between antiviral antibodies to multiple viruses and NPC.
• Provided new potential seromarkers for the diagnosis and prognosis of NPC.

## Graphical Abstract

**Figure:**
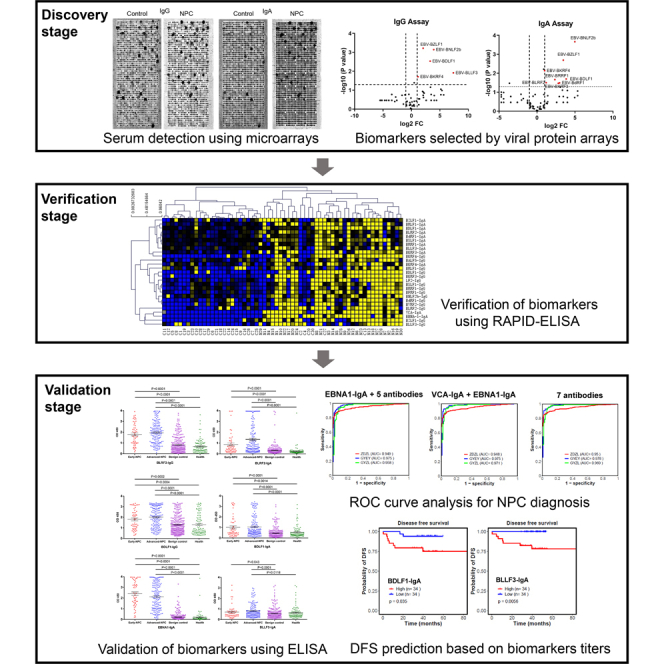

## In Brief

This study measured IgA and IgG antibody responses against 646 viral proteins from 23 viruses in nasopharyngeal carcinoma (NPC) patients and controls using nucleic acid programmable protein arrays. We found that five anti-Epstein-Barr virus (EBV) antibodies combined with the clinical biomarker EBV nuclear antigen 1 (EBNA1) IgA could be used to diagnose NPC accurately. Additionally, two anti-EBV antibodies showed potential as prognostic biomarkers of NPC. These results indicate the possibility of developing new serological tests for NPC detection and prognosis.

Viral infections are responsible for approximately 13% of human cancer development worldwide (1). Viruses that have been associated with cancer development in humans include Epstein-Barr virus (EBV), hepatitis B virus (HBV), hepatitis C virus (HCV), highly oncogenic human papillomaviruses (HPVs), human T-cell lymphotropic virus 1 (HTLV1), and human herpesvirus 8 (HHV8) (1, 2). Most virus-related cancers lack effective diagnosis, prognosis, and therapeutic targets. As such, reliable biomarkers could help bridge the gap between preventing and treating cancers that are caused by viruses (1).

Nasopharyngeal carcinoma (NPC), a malignant tumor originating from the nasopharyngeal mucosal lining, exhibits significant regional and ethnic differences with a high incidence in South China, Southeast Asia, and North Africa (3, 4, 5). Furthermore, it is important to note that NPC is extremely sensitive to radiation therapy, with a 5-years survival rate exceeding 90% in the early stages following radical radiotherapy. However, the overall survival rate for advanced NPC remains unsatisfactory (6, 7). Consequently, early screening and diagnosis of NPC play a pivotal role in enhancing patient prognosis.

Multiple lines of evidence have corroborated that the pathological process of NPC is intimately related to EBV infection (8). Moreover, high levels of anti-EBV antibodies have been detected in the blood of NPC patients, particularly IgA antibodies against viral capsid antigen (VCA), early antigen (EA), and EBV nuclear antigen 1 (EBNA1) (5, 9, 10, 11). These antibodies, especially the combination of VCA-IgA and EBNA1-IgA, are widely used to screen for and diagnose NPC (5, 9, 12, 13, 14, 15, 16, 17, 18).

Native VCA proteins are extracted from the pyrolysis products of human B lymphocytes infected with EBV and include capsid proteins such as VCA-p18 (BFRF3), VCA-p23 (BLRF2), and gp125/110 (BALF4) (16). ELISA kits that employ these native VCA proteins for detecting VCA-IgA have a higher specificity than ELISA kits that use recombinant VCA protein components (19). This may be because native VCA proteins have more antigenic epitopes than the recombinant VCA protein components (19). Methods that use recombinant proteins or peptide pools instead of native VCA proteins have been extensively explored to screen for or diagnose NPC. However, the proteins represented by these methods represent a tiny fraction of the ∼80 proteins that are expressed by the EBV genome. It is unclear whether antibodies targeting other EBV proteins will be effectively used for screening and diagnosis of NPC.

In addition to EBV, other viruses may play a role in the development of NPC, especially since many diseases are related to the interaction of multiple viruses (2, 20, 21). As an example, it has been demonstrated that type 1 diabetes (T1D) is associated with 14 different viruses and these viruses may contribute to the pathogenesis of T1D in at least two mechanisms (21). To date, only one report has hypothesized that HBV infection, apart from EBV infection, is related to increased NPC risk (22).

To help understand the role of viral infections in the development of NPC, we measured IgA and IgG antibody responses against 646 viral proteins from 23 cancer-related and other common viruses in patients with NPC and control subjects. Moreover, we validated the diagnostic value of candidate biomarkers for NPC by ELISA with more than 1600 serum samples from China Guangdong, which is one of the regions with the highest incidence of NPC in the world.

## Experimental Procedures

### Study Design and Patient Characteristics

**Fig. 1:**
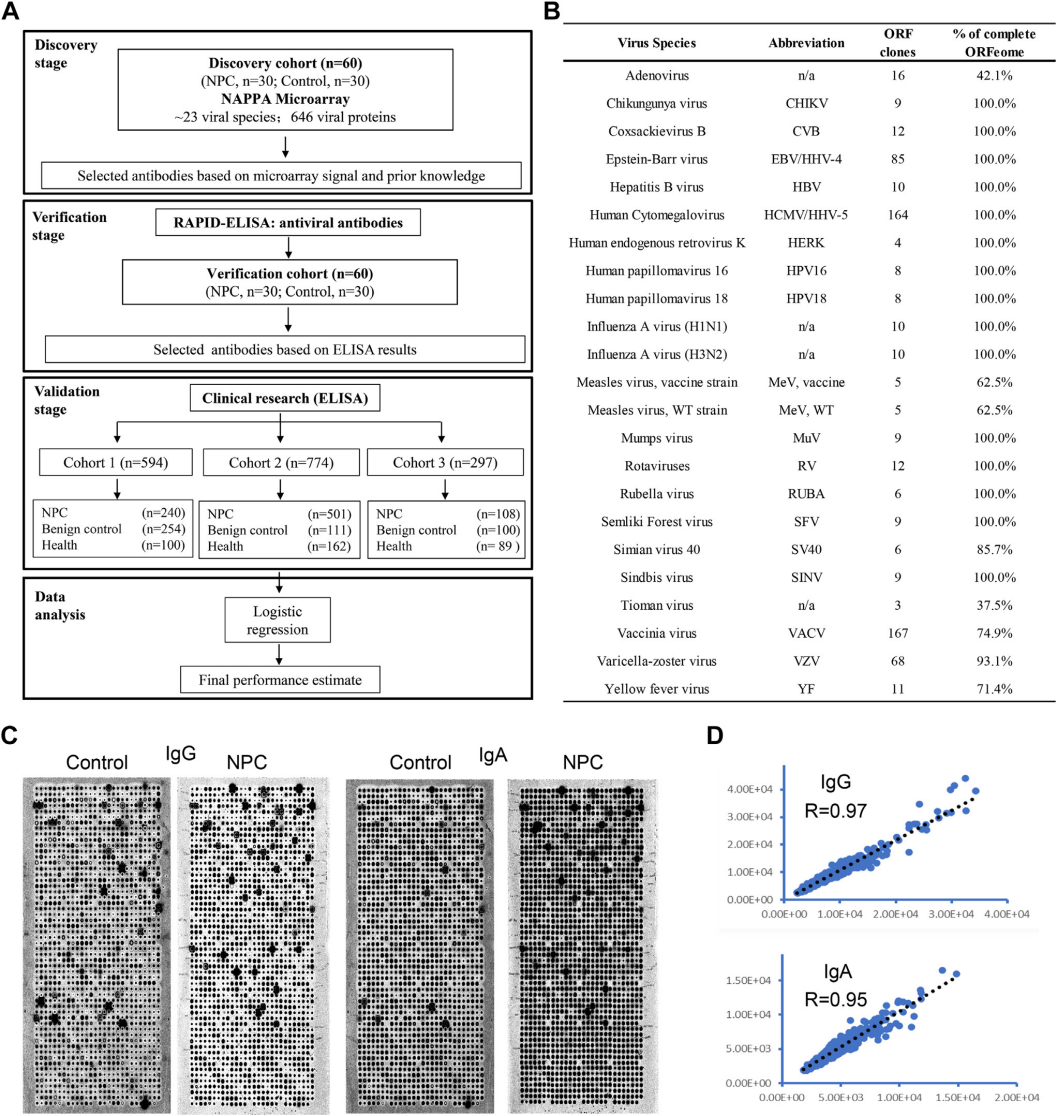
**Study flow chart, viral-NAPPA platform, and quality assessment.***A*, study design. In the discovery stage, we used NAPPA protein microarrays to display 646 viral proteins, which were then targeted by antibodies in 10 pooled NPC serum samples and 10 pooled control serum samples. In the verification stage, selected viral antibodies based on NAPPA data and prior knowledge were measured using RAPID-ELISA with 60 serum samples. Finally, statistically-significant predictive biomarkers were validated using a total of 1665 serum samples across three sample groups by ELISA. *B*, characteristics of the viral antigens, including the names of viral species, abbreviations, the number of ORF clones, and the percentage of complete ORFeome. n/a, not applicable. *C*, representative results of measured IgA and IgG antibody responses against 646 viral proteins from 23 viruses using NAPPA microarrays with the pooled serum from NPC patients and controls. *D*, scatter plot and correlation coefficients of serum antibody IgG and IgA detection on two slides from two serum screening days.

The study was conducted in three consecutive stages: discovery, verification, and validation (Fig. 1*A*). NAPPA arrays were employed in the discovery stage to display 646 viral proteins from 23 viruses to identify antibody profiles in 60 serum samples (Supplemental Table S1). In the second stage, the rapid antigenic protein *in situ* display ELISA (RAPID-ELISA) was used to verify the levels of selected viral antibodies in the same samples used in the discovery stage. Finally, viral antibody biomarkers were validated in three cohorts (Cohorts 1–3) containing 1665 serum samples (Supplemental Tables S2–S4) by ELISA.

Serum samples were collected from three hospitals in Guangdong, China, and stored at −80 °C. The sample set used for discovery and verification comprised of 30 NPC patients and 30 controls (Supplemental Table S1). Each pooled sample was prepared by mixing equal volumes of three NPC or control samples together, resulting in a total of 10 pooled NPC samples and 10 pooled control samples for the discovery stage using NAPPA. The 60 samples were analyzed individually with RAPID-ELISA in the verification stage.

For the validation stage, 1665 serum samples (Supplemental Tables S2–S4) from patients with NPC, patients with benign nasopharyngeal diseases, or patients presenting to otolaryngology with uncomfortable symptoms (benign control), and healthy people (health or healthy control) were tested. The samples were then divided into three cohorts (Cohorts 1–3) based on the hospital where the serum samples from patients with NPC were obtained: GYER (Cohort 1), ZDZL (Cohort 2), and GYZL (Cohort 3). The sample information is shown in Supplemental Tables S2–S4.

This study was conducted in accordance with the Declaration of Helsinki and was approved by the Ethics Committee of the Sun Yat-Sen University Cancer Center (Approval No. B2022-314-01), the second hospital of Guangzhou Medical University (Approval No.2017-hs-19), and the cancer hospital of Guangzhou medical university (Approval No. 2022-11).

### Pan-Cancer Viral Protein Microarray Testing

The pan-cancer viral protein (Fig. 1*B*) microarrays with good printing quality were designed and fabricated as previously described (23). Briefly, on each slide, two copies of every viral gene were printed. To demonstrate the successful detection of the secondary antibody, human IgA, IgM, and IgG were printed. Negative controls included empty spots, spots printed with printing buffer containing no plasmid DNA, and spots printed with printing buffer containing plasmid encoding haemagglutinin fusion protein and anti-GST capture antibody.

The protein microarrays expressing 646 viral antigens from 23 viruses (Fig. 1*B*) were prepared as previously described by using a human HeLa cell lysate–based protein expression system (Thermo Fisher Scientific) (23). Slides were first blocked using SuperBlock Blocking Buffer (Thermo Fisher Scientific) for 1 h at room temperature (RT) on a shaker. Then slides were washed with deionized water and dried by centrifugation at RT. Slides were sealed to prevent evaporation with a HybriWell (Grace Bio-Labs) and incubated with a HeLa cell lysate–based protein expression system at 30 °C for 1.5 h and 15 °C for 30 min.

To perform viral antibody screening, the expressed slides were blocked with 5% milk in phosphate-buffered saline with 0.2% Tween-20 (PBST) for 1 h, and incubated with diluted serum in 5% milk PBST at 4 °C overnight. After washing with PBST, slides were incubated with Cy3 donkey anti-human IgG antibody and Alexa Fluor 647 labeled rabbit anti-human IgA antibody for 1 h at RT. Slides were washed and dried. The fluorescence signal was detected and analyzed using the GenePix 4300A microarray scanner (Molecular Devices) and GenePix Pro7 software (Molecular Devices), respectively.

The reactivity of each antiviral antibody was graded by the intensity and morphology of spots with a “Halo ring” according to the previously described method (24, 25). The scores of the “Halo ring” of each viral protein from microarrays incubated with NPC patients’ serum (N) or control serum (C) were calculated. The potential biomarkers were selected according to the score ratio (N/C) ≥ 2 and a significant difference in score between the NPC group and control groups (*p*-value, *p* < 0.05).

### Rapid Antigenic Protein *In Situ* Display ELISA (RAPID-ELISA)

Candidate biomarkers were analyzed with RAPID-ELISA as previously described with minor modifications (23, 25). 96-well ELISA plates (Corning Inc) were coated with anti-GST antibody (GE Healthcare) at 10 ng/μl in coating buffer (sodium bicarbonate buffer, pH 9.6) overnight at 4 °C. The next day, coated plates were washed with PBST and blocked with 5% milk in PBST for 2 h. In the meantime, the antigens were expressed by incubating viral protein-encoding plasmid with the HeLa cell lysate–based protein expression system for 1.5 h at 30 °C. The plates were then incubated with the diluted viral antigens for 1 h at RT, washed again, and incubated with diluted serum for 1 h at RT. After washing with PBST, each well was incubated with horseradish peroxidase (HRP)-conjugated anti-human IgG or IgA antibody for 1 h at RT. Finally, the plates were washed and tetramethylbenzidine (TMB) substrate was added to the wells, and the reaction was stopped by adding 2 M sulfuric acid. After briefly shaking the plates, the absorbance at 450 nm was read.

### Expression of Potential Marker Antigens in Insect Cells

The genes of candidate marker antigens were amplified by polymerase chain reaction (PCR) using the plasmid DNA containing the candidate marker antigen gene as templates. Six histidine residues (His) were added at the N- and C-terminus of genes during PCR amplification. The target genes were cloned into the pFastBac1 vector (Invitrogen) and confirmed by sequencing. The recombinant bacmid was generated using the Bac-to-Bac System (Invitrogen). The recombinant baculovirus was generated by transfecting the recombinant bacmid to Sf9 cells using TransIT-Insect Transfection Reagent (Mirus Bio). The recombinant proteins that were extracted from baculovirus-infected cells were then analyzed by SDS-PAGE and western blotting (WB). Finally, the proteins were purified using a His-tagged protein purification kit (CoWin Biosciences).

### Expression of Potential Marker Antigens in *Escherichia coli*

The target genes amplified by PCR were cloned into the pGEX-4T-2 vector and confirmed by sequencing. Then the recombinant plasmids were transformed into *E. coli* DE3 competent cells and the expression of viral antigens was induced by isopropyl-β-D-thiogalactoside (IPTG). The expressed viral antigens were analyzed by SDS-PAGE and WB, and purified using the GST-tag protein purification kit (Beyotime Biotechnology).

### The Validation of Candidate Antibody Markers by ELISA

96-well microplates (Corning) were coated with the expressed recombinant viral protein at 4 °C overnight and blocked with 5% skim milk in PBST for 1 h at 37 °C after washing. The plates were then incubated with the diluted serum for 1h at 37 °C and washed again. Each well was incubated with HRP-conjugated anti-human IgG or IgA antibody for 1 h at 37 °C. After washing with PBST, TMB substrate was added to the wells, and the reaction was stopped by adding ELISA Stop Solution (Solarbio). After briefly shaking the plates, the absorbance at 450 nm was read. Anti-EBV VCA-IgA antibodies in serum samples were tested with commercial ELISA kits (EUROIMMUN), which employed native VCA proteins as antigens.

### Statistical Analysis

Antibody responses were visualized as a heatmap using the MultiExperiment Viewer software. The Mann–Whitney test was used to identify significantly produced antibodies to individual viral proteins between NPC and control groups in ELISA assays. The logistic regression model was established using the ELISA results of Cohort 2 (ZDZL) as the training set and the ELISA results of Cohort 1 (GYEY) and Cohort 3 (GYZL) as the test set. The sensitivity and specificity of the model to classify NPC cases were then evaluated by the receiver operating characteristic (ROC) curve analysis. The disease-free survival (DFS) of NPC patients was analyzed using the Kaplan-Meier method. A log-rank test was used to assess the significance of differentially expressed antibodies identified during the DFS analyses.

## Results

### Pan-Viral Proteome Analysis of Antibody Responses in Patients With NPC and Controls Using NAPPA-Based Microarrays

The overall study design is shown in Figure 1*A*. In the discovery stage, IgA and IgG antibody responses to 646 viral proteins from 23 viruses (Fig. 1*B*) were measured using NAPPA microarrays with pooled serum from patients with NPC or controls (Fig. 1*C*). The correlation coefficients of serological IgG and IgA detection on two slides across two serum screening days were 0.97 and 0.95, respectively (Fig. 1*D*).

**Fig. 2:**
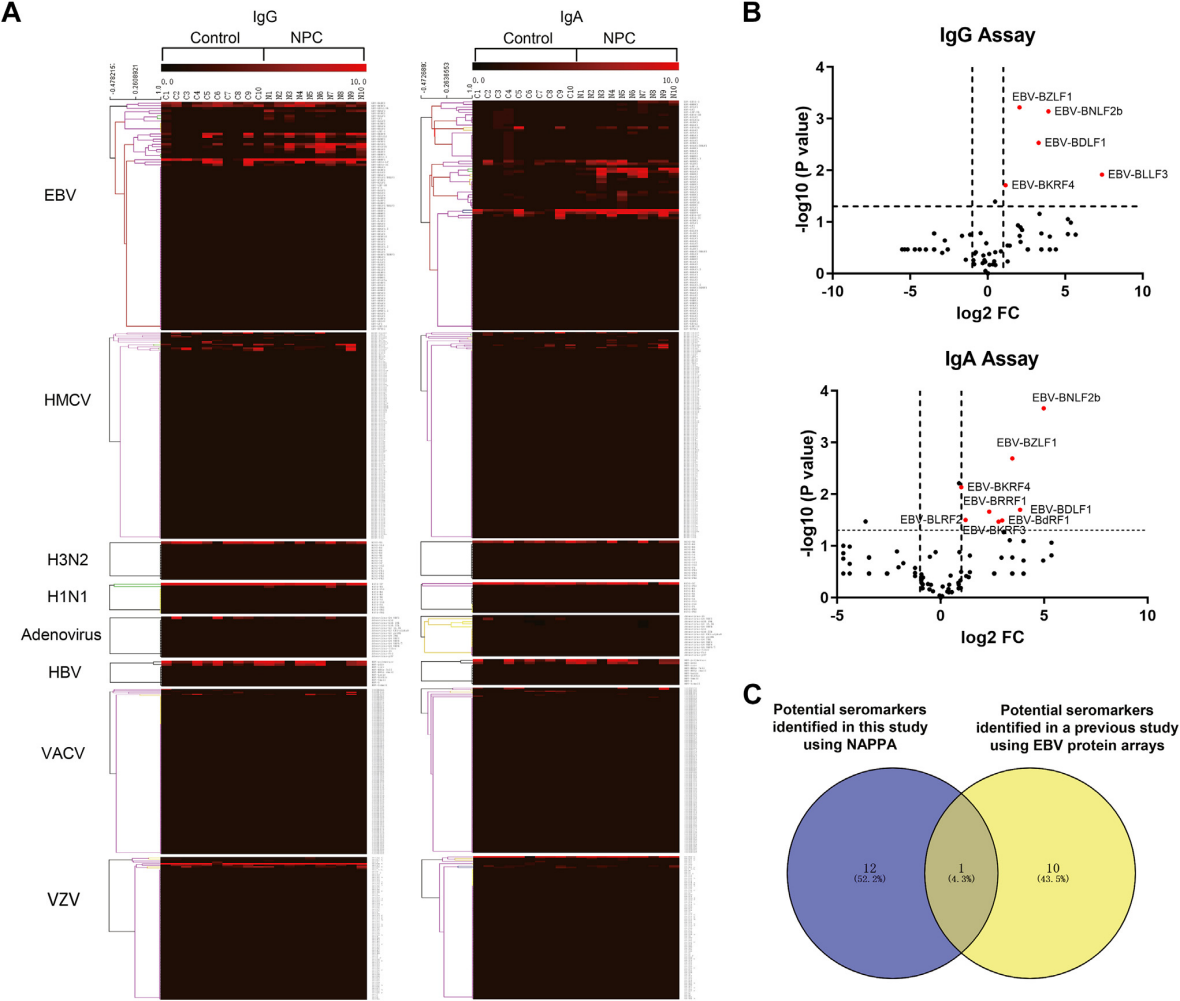
**Antibody profiling using viral protein arrays.***A*, heat maps of IgG and IgA reactivity to viral proteins from eight viruses. *B*, EBV-related antibodies selected by viral protein arrays. *C*, the promising EBV-related antibody targets for NPC prediction, identified from viral protein arrays in this study and a previous study.

We compared the virus-specific humoral immune responses between the NPC and the control groups. Heatmaps of IgG and IgA reactivity to viral proteins were generated using the scores of the “Halo ring” (Fig. 2*A*). IgG and IgA antibody responses to some proteins from human cytomegalovirus (HCMV), influenza A virus (H1N1 and H3N2), hepatitis B virus (HBV), vaccinia virus (VACV), and varicella-zoster virus (VZV) were detected in many pooled serum samples in both NPC and control groups (Fig. 2*A*), which suggests these proteins are highly immunogenic. Notably, we found some specific antibody responses to EBV antigens in NPC patients (Fig. 2*A*). Thirteen anti-EBV antibodies (BLRF2-IgA, BdRF1-IgA, BZLF1-IgA, BZLF1-IgG, BKRF4-IgA, BKRF4-IgG, BNLF2b-IgA, BNLF2b-IgG, BLLF3-IgG, BDLF1-IgA, BDLF1-IgG, BKRF3-IgA, BRRF1-IgA) were selected for further evaluation (Fig. 2*B*). BZLF1-IgG was one of the EBV-related antibody targets for NPC prediction, identified from custom protein microarrays in another previous study (6) (Fig. 2*C*).

### Verification of Selected anti-EBV Antibodies using RAPID-ELISA

**Fig. 3:**
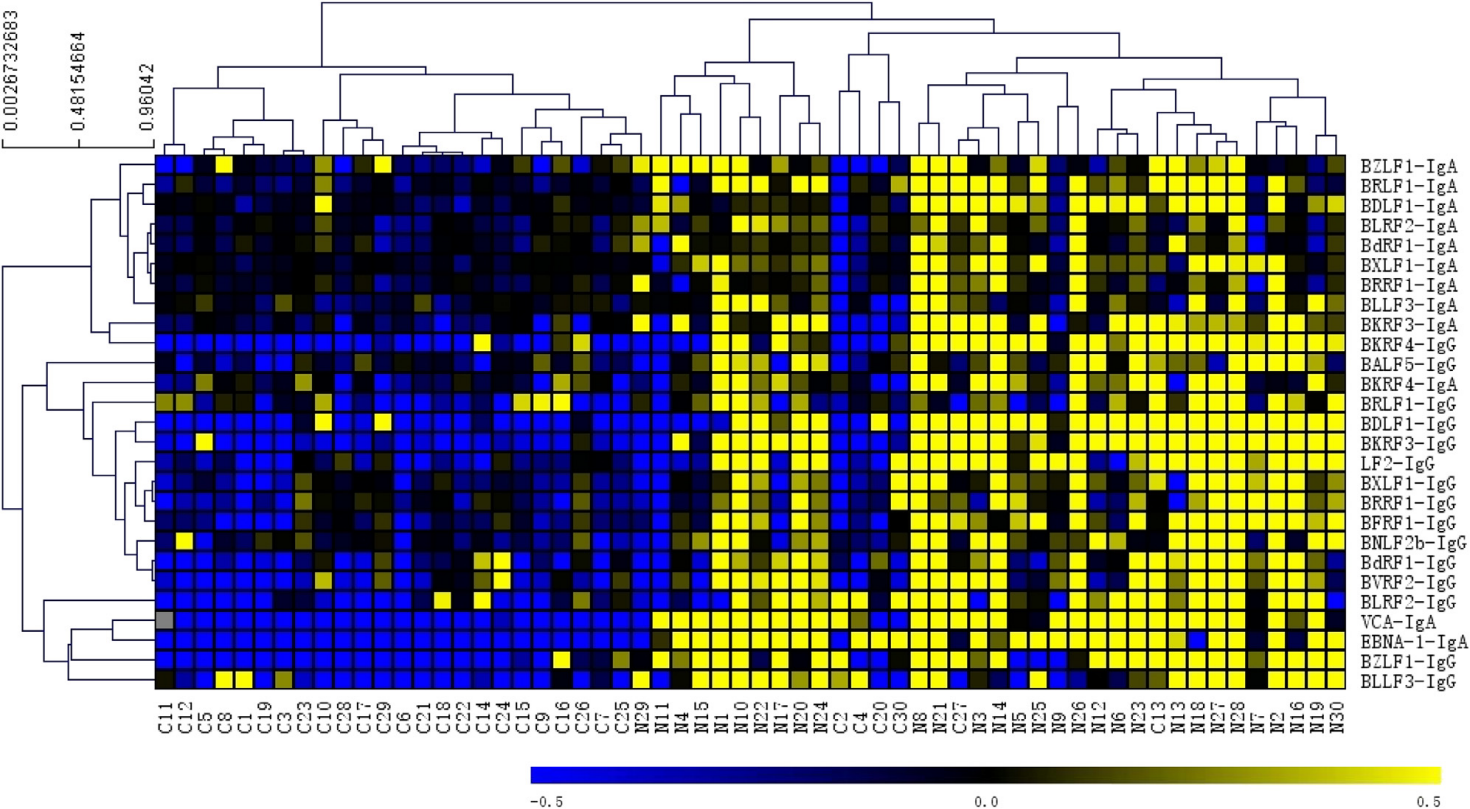
**Verification of selected anti-EBV antibodies using RAPID-ELISA.** Hierarchical clustering analysis of anti-EBV antibodies from patients with NPC and controls (*p* < 0.01).

For verification, we used RAPID-ELISA to measure 38 anti-EBV antibodies in 60 serum samples. These 38 antibodies included the 13 antibodies selected in the discovery stage and 25 antibodies identified in the pooled serum from NPC patients or controls using NAPPA microarrays or selected based on prior knowledge. In addition, we measured the EBV VCA-IgA antibody in serum samples using a commercial ELISA kit (EUROIMMUN). To better reveal the relationship between these anti-EBV antibodies and NPC, hierarchical clustering was performed. Significant difference in levels of 27 antibodies to 17 EBV proteins between patients with NPC and controls (*p* < 0.01) (Fig. 3). These results suggested that these antibodies may be potential seromarkers associated with NPC.

### Validation of Diagnostic Values of Seromarkers Using ELISA

To address the need for large-scale clinical sample testing, candidate marker antigens were expressed using the baculovirus or *E. coli* expression system. We successfully expressed and purified six EBV proteins: BLRF2, BDLF1, EBNA1, BLLF3, BRRF1, and LF2. The antibody levels to these proteins were determined by ELISA using a partial sample of NPC patients and benign controls in Cohort 1. Among these seromarkers, the antibody levels of EBV-BRRF1-IgG, and EBV-LF2-IgG were not significantly different between NPC samples and controls (*p* > 0.05, data not shown), and were not analyzed in additional samples.

**Fig. 4:**
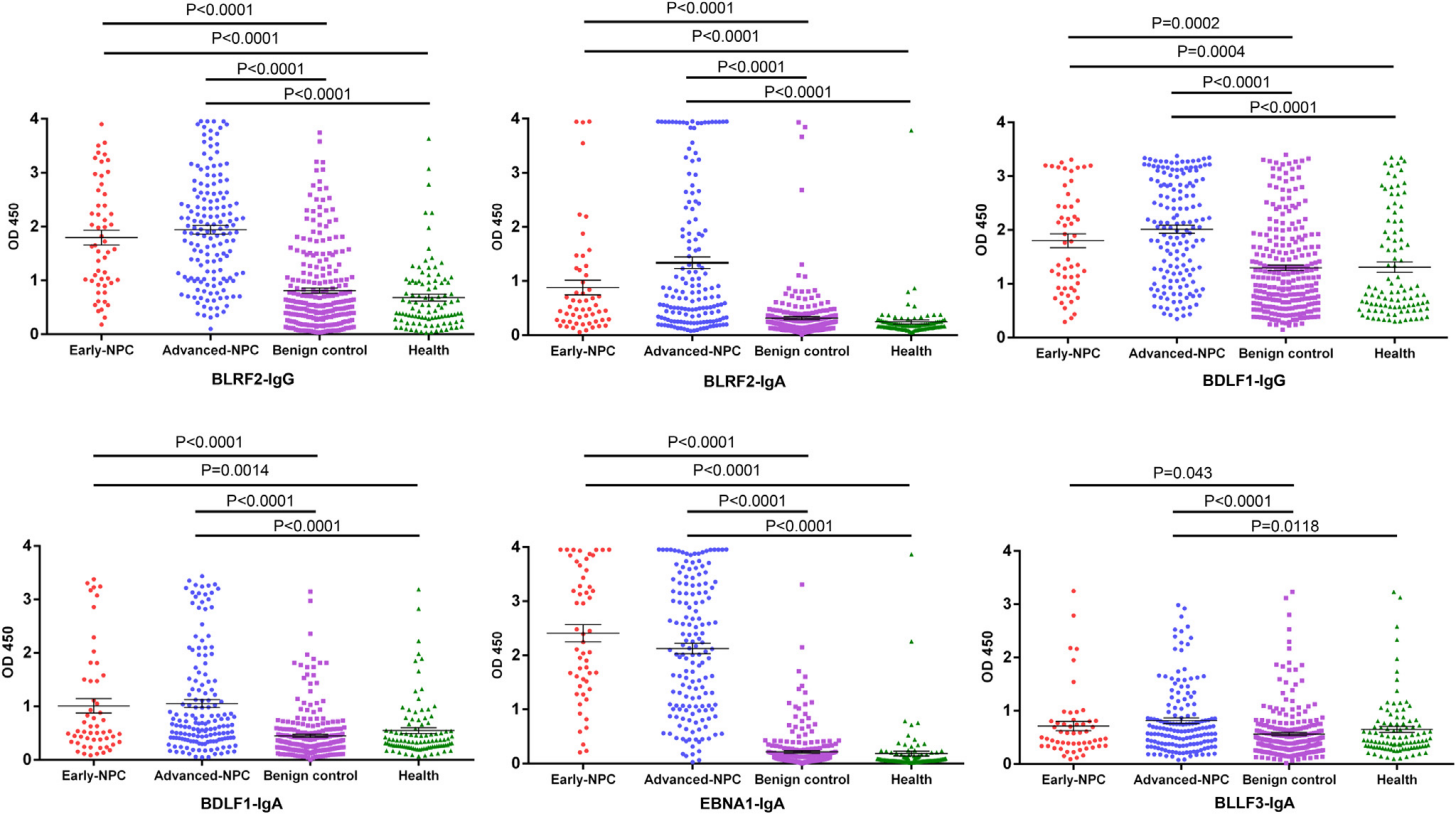
**Validation of selected anti-EBV antibodies using ELISA.** The antibody levels of six seromarkers (BLRF2-IgG, BLRF2-IgA, BDLF1-IgG, BDLF1-IgA, EBNA1-IgA, BLLF3-IgA) in serum samples from Cohort one were detected by ELISA.

Six antibodies (BLLF3-IgA, BLRF2-IgA, BLRF2-IgG, BDLF1-IgA, BDLF1-IgG, and EBNA1-IgA) were further evaluated with 1665 serum samples in three validation cohorts by ELISA. With the exception of BLLF3-IgA levels in early-stage NPC patients and controls (Fig. 4 and Supplemental Fig. S1), levels of all other seromarkers were significantly different (*p* < 0.05) between NPC patients (early-stage and advanced-stage) and controls in Cohort 1 (Fig. 4) and Cohort 2 (Supplemental Fig. S1). Also, levels of BLRF2-IgA, BLRF2-IgG, and EBNA1-IgA were significantly elevated in the NPC groups compared to controls (Supplemental Fig. S2). These seromarkers may play a role in the early diagnosis and differential diagnosis of NPC.

**Fig. 5:**
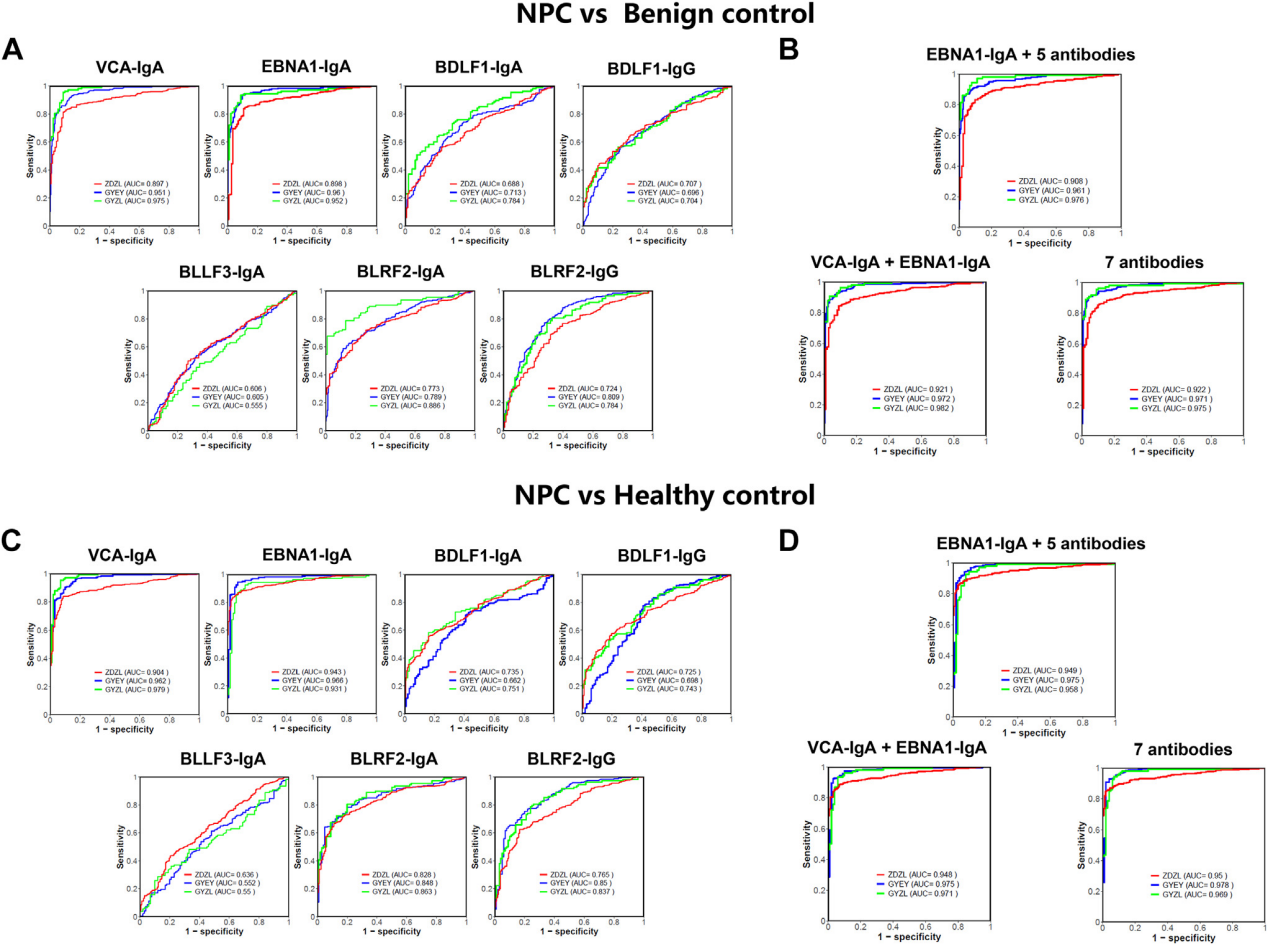
**Evaluation of candidate antibodies as diagnostic markers for NPC.***A* and *C*, ROC curve analysis for NPC diagnosis, by individual seromarker across three cohorts, respectively. *B* and *D*, ROC curve analysis for NPC diagnosis, by seromarkers combinations across three cohorts, respectively. GYEY represents Cohort 1; ZDZL represents Cohort 2; GYZL represents Cohort 3.

To further compare and analyze the role of these seromarkers in the diagnosis of NPC, we also tested levels of a known diagnostic marker of NPC, anti-EBV VCA-IgA, in serum from the three cohorts. Using the ELISA results of Cohort 2 (ZDZL) as the training set and the ELISA results of Cohort 1 (GYEY) and Cohort 3 (GYZL) as the test set to establish the logistic regression model (Fig. 5). At 95% specificity, the sensitivity of VCA-IgA to detect NPC ranged from 67% to 89% as determined by a ROC curve analysis (Table 1). The sensitivity of EBNA1-IgA ranged from 71% to 91% (Table 1). The other five seromarkers (BLLF3-IgA, BLRF2-IgA, BLRF2-IgG, BDLF1-IgA, and BDLF1-IgG) had limited ability to identify NPC patients when used individually (Fig. 5, *A* and *C*, Table 1).

**Table 1:** The sensitivity and specificity of antibody biomarkers of NPC

Seromarkers	The sensitivity at 95% specificity (NPC *versus* benign control)			The sensitivity at 95% specificity (NPC *versus* health)		
	Cohort 1	Cohort 2	Cohort3	Cohort 1	Cohort 2	Cohort3
VCA-IgA	81%	67%	81%	82%	73%	89%
EBNA1-IgA	82%	71%	84%	91%	85%	80%
BDLF1-IgA	22%	28%	41%	20%	37%	41%
BDLF1-IgG	20%	32%	29%	9%	35%	31%
BLLF3-IgA	12%	5%	6%	9%	16%	7%
BLRF2-IgA	41%	42%	68%	64%	47%	50%
BLRF2-IgG	25%	29%	25%	43%	34%	45%
EBNA 1-IgA +5 antibodies^a^	87%	76%	87%	92%	87%	85%
VCA-IgA + EBNA 1-IgA	90%	75%	91%	93%	86%	87%

a 5 antibodies is the combination of BLLF3-IgA, BLRF2-IgA, BLRF2-IgG, BDLF1-IgA, and BDLF1-IgG.

To simultaneously increase the specificity and sensitivity of the seromarkers in this study, ROC curve analyses of different combinations of seromarkers were generated using a logistic regression model (Fig. 5, *B* and *D*). We found that the sensitivity at 95% specificity of the 5-antibody (BLLF3-IgA, BLRF2-IgA, BLRF2-IgG, BDLF1-IgA, and BDLF1-IgG) plus ENBA1-IgA was similar to the sensitivity of two antibodies used to diagnose NPC in the clinic: VCA-IgA and ENBA1-IgA (Table 1). These results suggest that the combination of five antibodies (BLLF3-IgA, BLRF2-IgA, BLRF2-IgG, BDLF1-IgA, BDLF1-IgG) can replace the VCA-IgA, and combined with EBNA1-IgA can be effectively used in the diagnosis of NPC.

### Survival Analysis

**Fig. 6:**
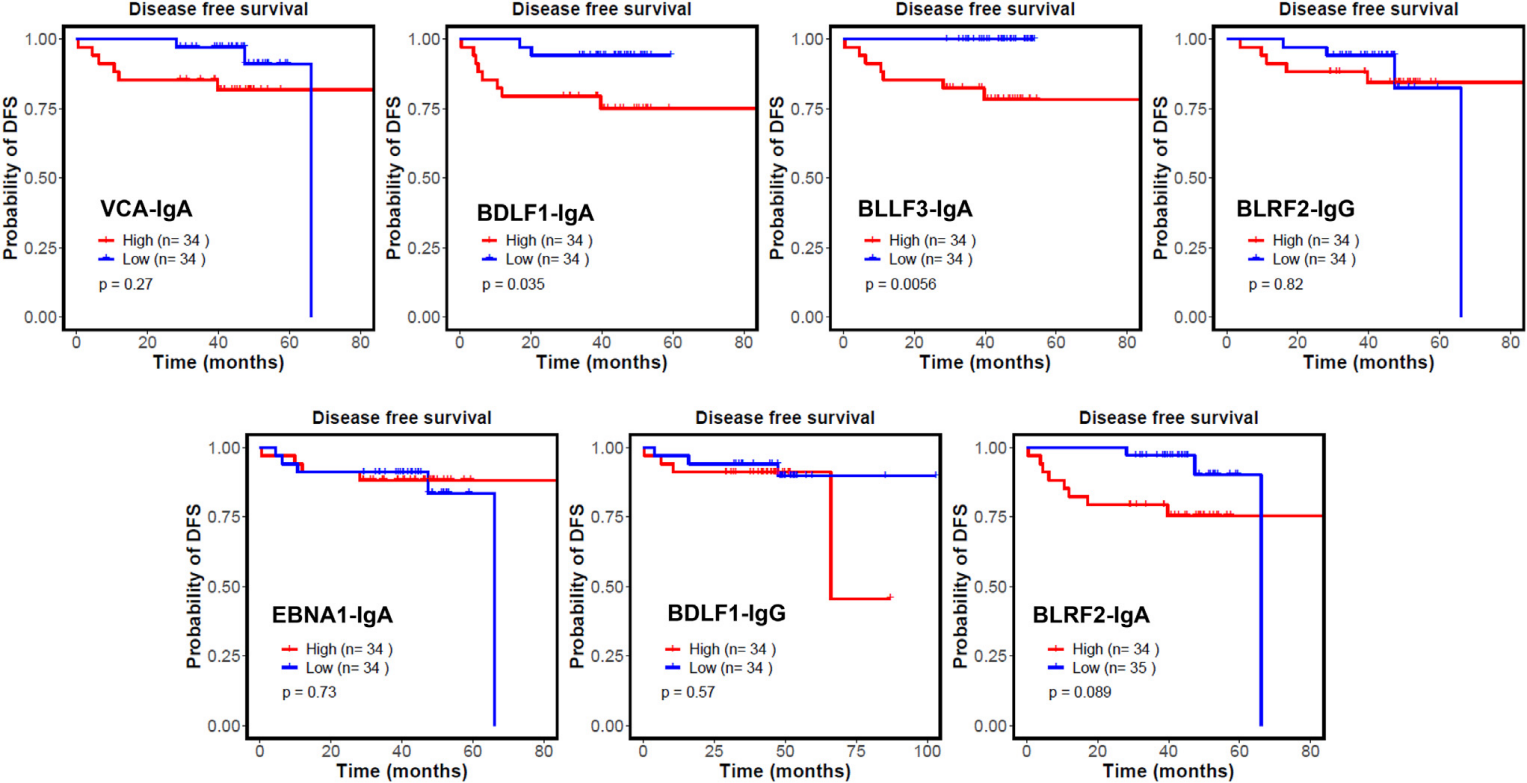
**DFS prediction basedon seromarker titers.**

We collected prognostic information from 136 patients in Cohort 1. The levels of BLLF3-IgA and BDLF1-IgA were significantly (*p* < 0.05) associated with the probability of disease-free survival (DFS) (Fig. 6), as determined with Kaplan-Meier method estimates. However, the survival curves of other seromarkers showed no significant relationship between antibody levels and the probability of DFS (Fig. 6).

## Discussion

NAPPA arrays overcome many of the limitations of traditional protein microarrays (26). It avoids the tedious process of protein preparation and purification, in which hundreds to thousands of proteins are expressed *in situ* for high-throughput rapid analysis within hours (23, 26, 27). High-throughput viral proteome microarrays based on NAPPA technology have been previously applied to study the correlation between viral infection and disease (6, 20, 23, 27). In this study, we measured both IgA and IgG antibody responses against 646 viral proteins from 23 viruses in patients with NPC and control subjects using NAPPA, identifying new candidate biomarkers for NPC diagnosis in serum. To our knowledge, this is the first study using NAPPA to examine the association between antiviral antibodies to multiple viruses and NPC. IgM antibody (*e.g.* VCA IgM) usually appears at the early stage of EBV acute infection, but disappears completely within a few weeks (28). Thus far, at least 35 antibodies against EBV proteins have been reported for diagnosis and screening of NPC. The antibody types for these biomarkers were all IgA or IgG, with no IgM (29). IgM antibodies against EBV proteins may not be potential diagnostic markers for NPC, so we did not screen the IgM antibody profile in this study.

A large number of antibody responses to different viral antigens in NPC and control groups were detected in our study, but only a higher EBV-specific immune response was found in NPC (Fig. 2, *A* and *B*). This reflects the advantages of NAPPA-based viral proteomic microarrays in terms of rapidity, high sensitivity, and high throughput of detection. Our test results in this study are consistent with the previous reports indicating the strong association between NPC and EBV infection (3, 30). To verify the diagnostic values of these potential seromarkers for NPC, more than 1600 serum samples from three cohorts were tested by ELISA in this study. We found that the antibody levels of six seromarkers (BLRF2-IgA, BLRF2-IgG, BDLF1-IgA, BDLF1-IgG, EBNA1-IgA, BLLF3-IgA) in patients with NPC were significantly higher than those in the benign controls and healthy controls, and most of these antibodies were also elevated in the serum of patients with early-stage NPC (Fig. 4, Supplemental Figs. S1 and S2). BLRF2 is a tegument protein that is closely associated with the EBV capsid and is called viral capsid antigen (VCA)-p23 in immunological studies (31, 32). Previous studies have shown that ELISAs that used recombinant VCA-p18 and VCA-p23 for analysis could be effectively used for serological diagnosis of EBV infection (33). BDLF1 is the minor capsid protein of EBV and forms a triplex with BORF1 (34). The interaction of the EBV triplex with VCA is important for the assembly of the EBV capsid (34). The BLLF3 gene encodes a deoxyuridine triphosphate nucleotidohydrolase (dUTPase), which modulates innate and adaptive immune responses through the involvement of the Toll-Like Receptor 2 (TLR2) (35). It has been demonstrated that serum samples from patients with diffuse large B-cell lymphoma and chronic lymphocytic leukemia exhibit increased anti-dUTPase antibodies (35).

To further determine whether these seromarkers could accurately diagnose NPC, a logistic regression model was established using the ELISA results. None of the seromarkers by themselves could identify NPC patients accurately (Fig. 5, *A* and *C*, Table 1). Therefore, models using different antibody combinations were built (Fig. 5, *B* and *D*). A combination of ENBA1-IgA with five other antibodies (BLLF3-IgA, BLRF2-IgA, BLRF2-IgG, BDLF1-IgA, BDLF1-IgG) resulted in a similar sensitivity at 95% specificity to diagnose NPC as the known biomarker combination, VCA-IgA and ENBA1-IgA (Table 1). Importantly, ELISAs that employ the recombinant proteins (BLLF3, BLRF2, BDLF1) are easier to produce and standardize than VCA-based ELISAs that use native VCA proteins.

The ability of seromarker titers to predict the outcome of NPC patients was also examined. In line with previous studies (36, 37), our results demonstrate that antibody titers of known seromarkers, VCA-IgA and EBNA1-IgA, could not be used to predict the survival of NPC patients accurately. However, NPC patients with elevated levels of BLLF3-IgA and BDLF1-IgA titers had significantly worse DFS, suggesting these two seromarkers may be potential prognostic indicators of NPC (Fig. 6). It is also possible that these proteins play a role in the development and progression of NPC, and could be potential targets for NPC therapy.

There are several limitations to our study. First, some viral proteins could not be successfully expressed and purified. Second, the number of samples used for the survival analysis is low. The potential biomarkers BLLF3-IgA and BDLF1-IgA for predicting outcomes of patients with NPC should be further verified with multi-center studies and larger sample cohorts in the future.

This study measured IgA and IgG antibody responses against 646 viral proteins from 23 viruses in NPC patients and controls using NAPPA. We found that five anti-EBV antibodies (BLLF3-IgA, BLRF2-IgA, BLRF2-IgG, BDLF1-IgA, BDLF1-IgG) combined with the the clinical biomarker EBNA1-IgA could be used to diagnose NPC accurately. In addition, two of the seromarkers, BLLF3-IgA and BDLF1-IgA, could be potential prognostic biomarkers of NPC. These results indicate the possibility of developing new serological tests for NPC detection and prognosis.

## Data Availability

Most data supporting the findings are provided within the manuscript and in the supplemental data. Original data are available from the corresponding author on reasonable request.

## Supplemental data

This article contains supplemental data.

## Conflict of interest

All authors declare no competing interests.
