# Supplementary material for: Antibody Profiling of Pan-Cancer Viral Proteome Reveals Biomarkers for Nasopharyngeal Carcinoma Diagnosis and Prognosis
Source: Mol Cell Proteomics. 2024 Feb 1;23(3):100729. doi: 10.1016/j.mcpro.2024.100729 (PMC10933552; doi:10.1016/j.mcpro.2024.100729)
Supplement: Supplemental Data [file mmc1.docx]

-Supplementary Information-

**This file includes:**

**Figures S1 and S2**

**Tables S1 to S4**

**Supplementary Figures**


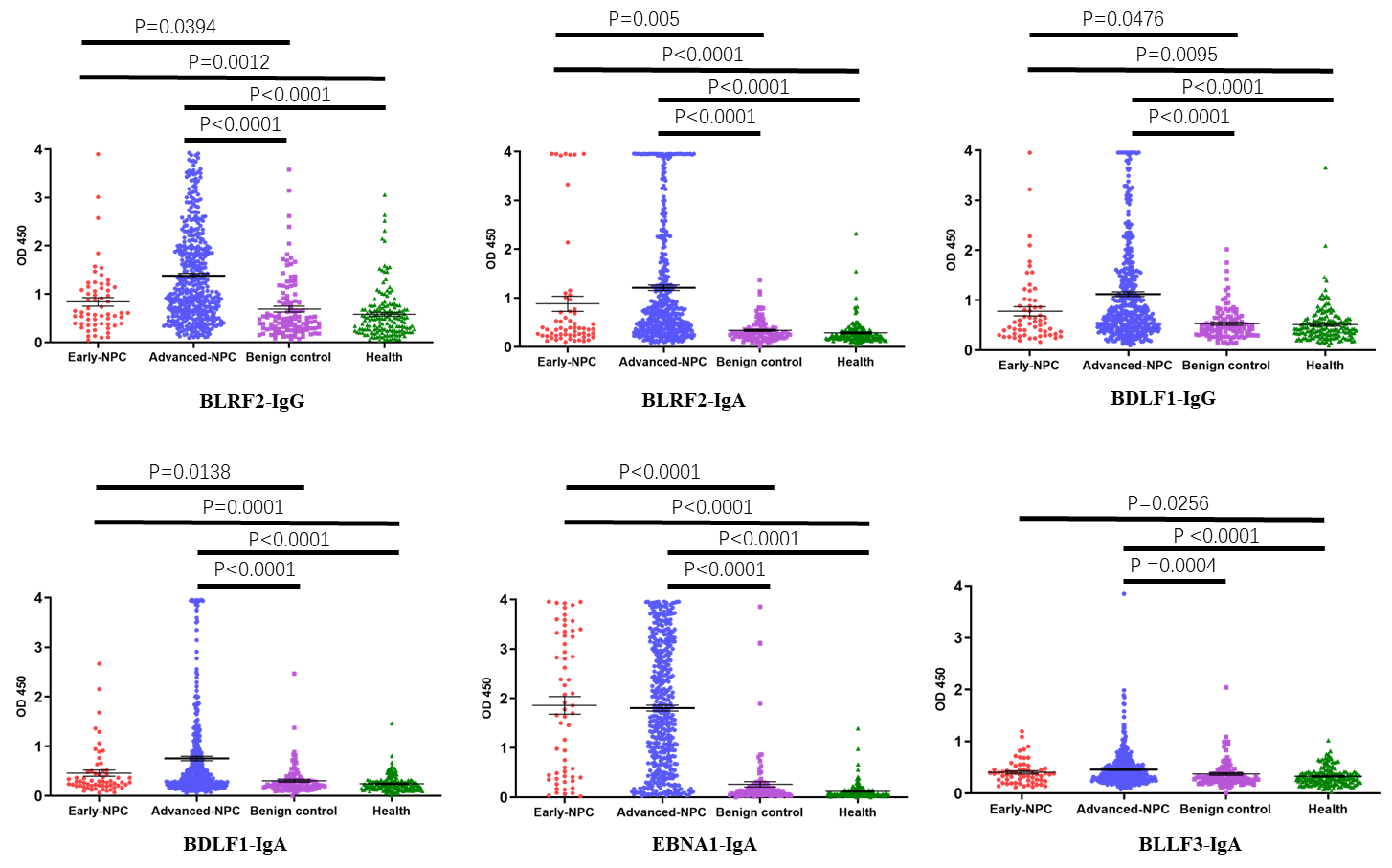


**Figure S1. Validation of selected** **anti-EBV antibodies using ELISA in Cohort 2.** The antibody levels targeting 6 seromarkers (BLRF2-IgG, BLRF2-IgA, BDLF1-IgG, BDLF1-IgA, EBNA1-IgA, BLLF3-IgA) in Cohort 2 were detected by ELISA, respectively.


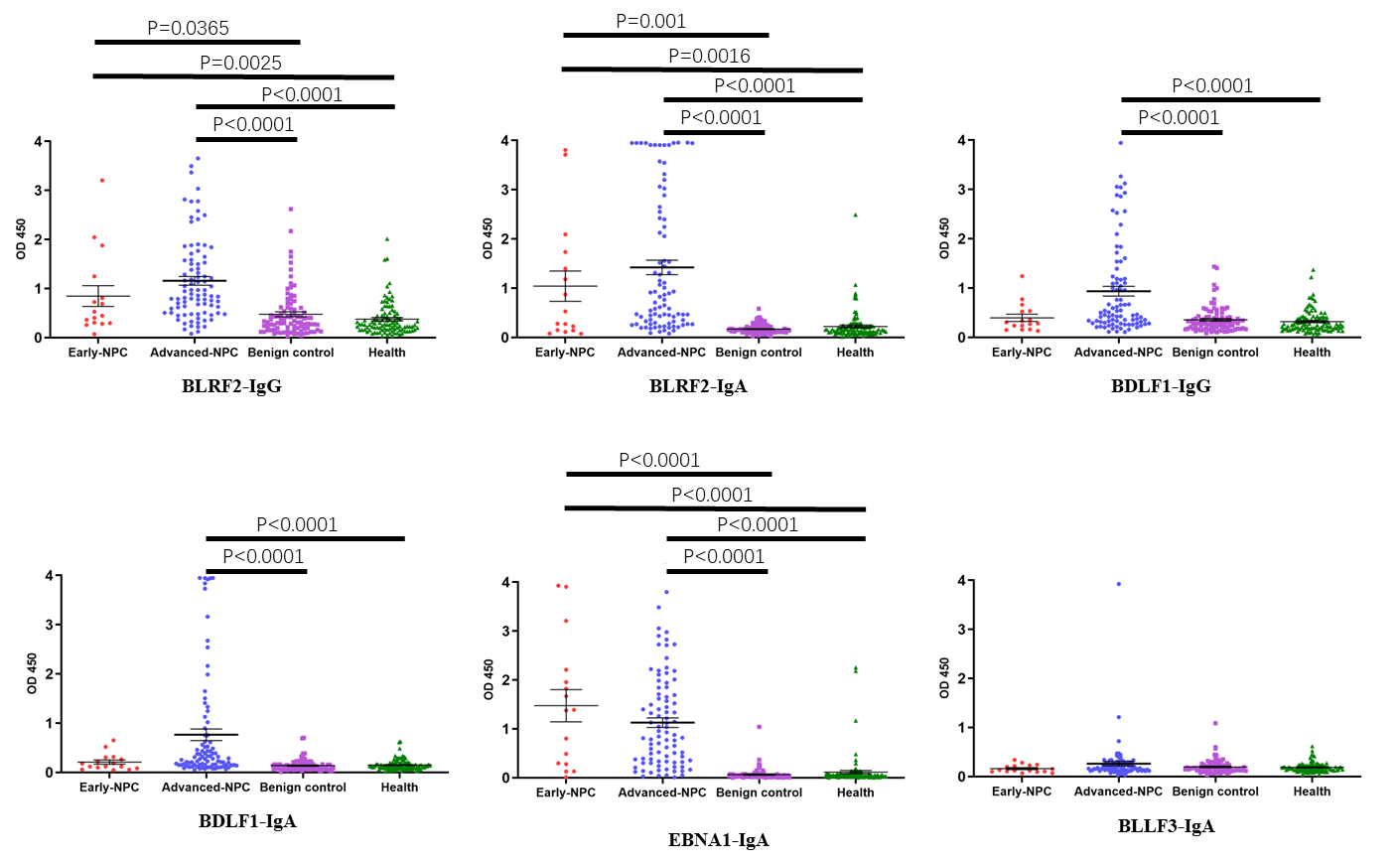


**Figure S2. Validation of selected anti-EBV antibodies using ELISA in Cohort 3.** The antibody levels targeting 6 seromarkers (BLRF2-IgG, BLRF2-IgA, BDLF1-IgG, BDLF1-IgA, EBNA1-IgA, and BLLF3-IgA) in Cohort 3 were detected by ELISA, respectively.

**Supplementary Tables**

**Table S1.** **Clinical samples information in the discovery stage and verification stage**

|  | NPC (n=30) | Control subjects (n=30) |
| --- | --- | --- |
| Age, mean (SD) | 49.1 (8.0) | 49.4 (7.9) |
| Age, range in years | 30-61 | 32-61 |
| Sex |  |  |
| Male | 20 (66.67%) | 20 (66.67%) |
| Female | 10 (33.33%) | 10 (33.33%) |
| Clinical stage |  |  |
| Early stage | 0 |  |
| Advanced stage | 30 |  |
| Unknown | 0 |  |

**Table S2.** **Clinical information of Cohort 1 samples employed for validation**

| Cohort 1, n=594 | | | | | |  |
| --- | --- | --- | --- | --- | --- | --- |
|  | NPC (n=240) | Benign Control (n=254) | | Health (n=100) |  |  |
| Age, mean (SD) | 51.6 (12.2) | 50.9 (12.1) | | 50.22 (10.0) | | |
| Age, range in years | 20-84 | 21-93 | | 24-76 | | |
| Sex |  |  | |  | | |
| Male | 174 (72.5%) | 178 (70.08%) | | 71 (71%) | | |
| Female | 66 (27.5%) | 76 (29.92%) | | 29 (29%) | | |
| Clinical stage |  |  | |  | | |
| Early stage | 54 |  | |  | | |
| Advanced stage | 156 |  | |  | | |
| Unknown | 30 |  | |  | | |
|  |  |  |  | | |  |

**Table S3.** **Clinical information of Cohort 2 samples employed for validation**

| Cohort 2, n=774 | | | | | |  |
| --- | --- | --- | --- | --- | --- | --- |
|  | NPC (n=501) | Benign Control (n=111) | | Health (n=162) |  |  |
| Age, mean (SD) | 44.9 (11.3) | 52.1 (14.9) | | 51.2 (17.8) | | |
| Age, range in years | 12-77 | 20-84 | | 22-85 | | |
| Sex |  |  | |  | | |
| Male | 351 (70.1%) | 58 (52.3%) | | 67 (41.4%) | | |
| Female | 150 (29.9%) | 53 (47.7%) | | 95 (58.6%) | | |
| Clinical stage |  |  | |  | | |
| Early stage | 61 |  | |  | | |
| Advanced stage | 433 |  | |  | | |
| Unknown | 7 |  | |  | | |
|  |  |  |  | | |  |

**Table S4.** **Clinical information of Cohort 3 samples employed for validation**

| Cohort 3, n=297 | | | | | |  |
| --- | --- | --- | --- | --- | --- | --- |
|  | NPC (n=108) | Benign Control (n=89) | | Health (n=100) |  |  |
| Age, mean (SD) | 51.8 (13.8) | 51.7 (14.4) | | 51.4 (3.5) | | |
| Age, range in years | 19-89 | 17-86 | | 44-59 | | |
| Sex |  |  | |  | | |
| Male | 81 (75%) | 41 (46.1%) | | 75 (75%) | | |
| Female | 27 (25%) | 48 (53.9%) | | 25 (25%) | | |
| Clinical stage |  |  | |  | | |
| Early stage | 16 |  | |  | | |
| Advanced stage | 88 |  | |  | | |
| Unknown | 4 |  | |  | | |
|  |  |  |  | | |  |
